# Longitudinal associations between adverse childhood experiences and moderate-risk to problem gambling in young adulthood: A prospective UK cohort study

**DOI:** 10.1016/j.abrep.2026.100764

**Published:** 2026-10-22

**Authors:** Emily Patterson, Raffaello Rossi, Hannah Sallis, Emma Dennie, Laura D. Howe, Alan Emond, Annie Herbert

**Affiliations:** a Public Health Training Programme, South West, UK; b Business School, University of Bristol, Bristol, UK; c MRC Integrative Epidemiology Unit, University of Bristol, Bristol, UK; d Centre for Academic Mental Health, University of Bristol, Bristol, UK; e Population Health Sciences, University of Bristol, Bristol, UK; f University of Kentucky School of Social Work, KY, United States; g Centre for Academic Child Health, University of Bristol, Bristol, UK

**Keywords:** ALSPAC, Gambling, Adverse childhood experiences, PGSI, Early life stresses, Adolescent trauma

## Abstract

Previous research links Adverse Childhood Experiences (ACEs) with problem gambling, but most studies rely on retrospective reporting and focus narrowly on maltreatment, overlooking adversities such as parental mental health issues. Using data on 3794 young adults in the Avon Longitudinal Study of Parents and Children, we examined longitudinal associations between 10 prospectively measured ACEs (individually and cumulatively), and being at least at moderate-risk of problem gambling (Problem Gambling Severity Index ≥ 3) at ages 17, 20 and 24, adjusted for socioeconomic and other background factors. Population attributable fractions (PAFs) estimated proportions of cases potentially attributable to ACEs. Most ACEs were associated with higher odds of moderate-risk/problem gambling across ages (25/30 estimates) after adjustment, though effect sizes were generally small (median adjusted odds ratio [aOR] 1.37, interquartile range 1.19–1.45), and confidence intervals (CIs) wide. Sexual abuse showed the strongest association (aORs 1.8–2.8, CIs 0.6–6.0), while bullying and parental conviction were associated at ages 17 and 20 only, parental separation age 24 only. Evidence for a dose-response relationship was weak. PAFs suggested ACEs accounted for up to 11% of moderate-risk/problem gambling cases. These findings highlight potential impacts of ACEs on later gambling behaviour, but imprecise estimates suggest findings should be interpreted cautiously and strengthened through larger sample sizes and meta-analyses.

## Highlights

• In one of the first prospective studies of the longitudinal association between ten different Adverse Childhood Experiences (ACEs) and gambling harms in young adulthood, several ACEs were positively associated with at least moderate-risk of problem gambling at three different ages, though estimates were imprecise.
• There was little evidence of a dose-response relationship between number of ACEs experienced and gambling risk.
• Population attributable fractions suggested that ACEs could account for a modest proportion of gambling harms (up to 11%).
• Findings indicate possible but unconfirmed links between ACEs and gambling harms, highlighting the need for further large-scale longitudinal research.

## 1 Introduction

Gambling is widespread internationally and is increasingly recognised as a public health concern. A recent meta-analysis of representative surveys from 68 countries and territories estimated that 46.2% of adults had gambled in the previous 12 months, 8.7% were classified as engaging in gambling carrying any risk of harm, and 1.41% in problem gambling (Tran et al., 2024). Most of the included samples were from high income countries, and the pooled estimates show substantial variation between countries and measurement approaches. The 2023 Gambling Survey for Great Britain estimated that 3.7% of adults scored between 3 and 7 on the Problem Gambling Severity Index (PGSI), indicating moderate risk of problem gambling, while 2.5% scored 8 or more, indicative of problem gambling (Gambling Commission, 2024). Gambling problems can be associated with financial, relationship, physical, mental health, and social harms (Emond & Griffiths, 2020;Gunstone & Gosschalk, 2020; Public Health England & Office for Health Improvement and Disparities, 2023). These harms can extend beyond the person who gambles, to their families and wider social networks (Gunstone & Gosschalk, 2020; Public Health England & Office for Health Improvement and Disparities, 2023).

Young adulthood may be a particularly important period in the development of gambling problems.^1^ In Great Britain, PGSI scores of 3 or more are more common among adults aged 18 to 34 than among older age groups (Gambling Commission et al., 2024). This period combines continuing development in self-regulation and decision making with increasing financial independence, greater access to gambling and extensive exposure to online gambling environments (Blakemore & Choudhury, 2006; Chambers & Potenza, 2003; Emond et al., 2020; Rossi & Nairn, 2024). Identifying factors that precede gambling problems during this developmental period is therefore important.

One potential early life factor is adverse childhood experiences (ACEs). ACEs are potentially harmful or stressful experiences occurring before the age of 18, including abuse, neglect, bullying, violence between parents, and household difficulties such as caregiver mental ill health, substance use, offending, or parental separation (Felitti et al., 1998). ACEs are common in most populations worldwide. A meta-analysis of 206 studies involving more than half a million adults across 22 countries estimated that approximately 60% had at least one type of ACE and 16.1% had four or more, although prevalence varied across populations and measurement approaches (Madigan et al., 2023). In a recent UK sample of people seeking treatment for gambling problems, 81% reported at least one type of ACE and 41% reported four or more (Moxey et al., 2026). ACEs have also been linked to a broad range of adverse health, social, and behavioural outcomes across the life-course, including smoking and substance misuse (Bellis et al., 2019; Felitti et al., 1998).

Several developmental mechanisms could plausibly link ACEs with later gambling problems. Proposed mechanisms include stress-related disruptions to emotional regulation and impulsivity (Bick & Nelson, 2016; Blakemore & Choudhury, 2006; Espeleta et al., 2018; Soares et al., 2021). Contemporary theories of gambling disorder suggest that some individuals engage in gambling as a means of escaping negative affect, managing psychological distress, or regulating emotions (Blaszczynski & Nower, 2002; Sharpe, 2002). The Pathways Model proposes an emotionally vulnerable pathway to gambling harms, in which adverse experiences and associated psychological difficulties may increase susceptibility (Blaszczynski & Nower, 2002). Gambling may therefore emerge as a maladaptive coping strategy for managing distress or seeking reward and stimulation following adversity (Dowling et al., 2017; Poole et al., 2017). ACEs may consequently contribute to gambling risk through developmental pathways that shape emotional functioning, coping styles, and responses to stress across the life-course.

Despite these plausible theoretical pathways and growing interest in understanding gambling from a developmental and life-course perspective (Russell et al., 2022), robust longitudinal evidence on ACEs and gambling is limited. Prior reviews have highlighted associations between child maltreatment and later problem gambling, but most available studies rely on cross-sectional or retrospective designs (Lane et al., 2016). More recent work examining broader stressful life events and family-level influences similarly suggests that early adversity may play a role in gambling vulnerability, but these studies still lack prospective childhood ACE measurement (Russell et al., 2022; Smith et al., 2025). These designs raise concerns about recall bias and temporal ambiguity, which limits our ability to assess developmental models that conceptualise ACEs as determinants of later vulnerability (Baldwin et al., 2019). Prospective cohort studies, in which ACEs are measured during childhood and gambling outcomes assessed subsequently, provide a stronger test of these developmental hypotheses and can help identify whether specific adversities or cumulative experiences are associated with later gambling risk.

Existing studies have also tended to focus narrowly on maltreatment rather than the full possible range of ACEs. It is therefore unclear whether any association reflects the cumulative burden of adversity or stronger associations with particular experiences (Evans et al., 2013). Dimensional models suggest that different forms of adversity may affect development through partly distinct processes, such as experiencing threat or deprivation (McLaughlin et al., 2014). Examining ACEs both individually and cumulatively could therefore help clarify whether later gambling problems are associated with overall adversity burden or with particular forms of childhood experience.

Policy discussions in the UK have similarly highlighted gaps in the evidence: the 2023 Gambling Act White Paper concluded that there was insufficient evidence for a strong association between child maltreatment and gambling harms (Public Health England & Office for Health Improvement & Disparities, 2023). A British public health evidence review concluded that child maltreatment may be associated with subsequent harmful gambling but emphasised the need for stronger longitudinal evidence. Stronger prospective evidence on ACE-gambling relationships could support prevention strategies, early identification, and trauma-informed care (Asmussen et al., 2020), particular as gambling-related care increasingly moves into the National Health Service's remit (National Institute for Health and Care Excellence, 2025). A prospective UK cohort therefore provides an opportunity to address an internationally- and policy- relevant evidence gap.

## 2 Aims and objectives

Using data from the Avon Longitudinal Study of Parents and Children (ALSPAC) we aimed to investigate the longitudinal associations between 10 different types of ACEs (as total number of ACEs and each ACE separately) measured during childhood, and moderate-risk or problem gambling in young adulthood at ages 17, 20, and 24 years. Our focus was on associations adjusted for measured background confounders, to estimate the causal contribution of ACEs to subsequent gambling risk.

The specific objectives were to investigate:

1. The longitudinal relationship between types of ACEs (individual and number of) and at least moderate-risk of problem gambling (a Problem Gambling Severity Index [PGSI] score of 3+).
2. The extent to which ACEs accounted for cases of moderate-risk or problem gambling in young adults.

Given published findings between ACEs and other addictive behaviours, we hypothesised that the adjusted associations between the number of ACEs and experiencing moderate-risk or problem gambling would follow a ‘dose-response’, where there were stronger associations for higher numbers of ACEs (Hughes et al., 2017).

## 3 Methods

### 3.1 The ALSPAC cohort and current study sample

Our study used data from ALSPAC, a UK-based birth cohort (www.bristol.ac.uk/alspac) (Boyd et al., 2013; Fraser et al., 2013; Northstone et al., 2019). Full details of recruitment, follow-up procedures and participant numbers are provided elsewhere and summarised in Supplementary Box S1. The study website contains details of all the data that are available through a fully searchable data dictionary and variable search tool (http://www.bristol.ac.uk/alspac/researchers/our-data/). We analysed data on the children of the cohort (the index offspring or ‘G1s’). Participants were eligible if they had data on at least one ACE measure and at least one PGSI assessment at age 17, 20, and/or 24 years. The analytical sample comprised 3794 participants (Fig. 1).

**Fig. 1:**
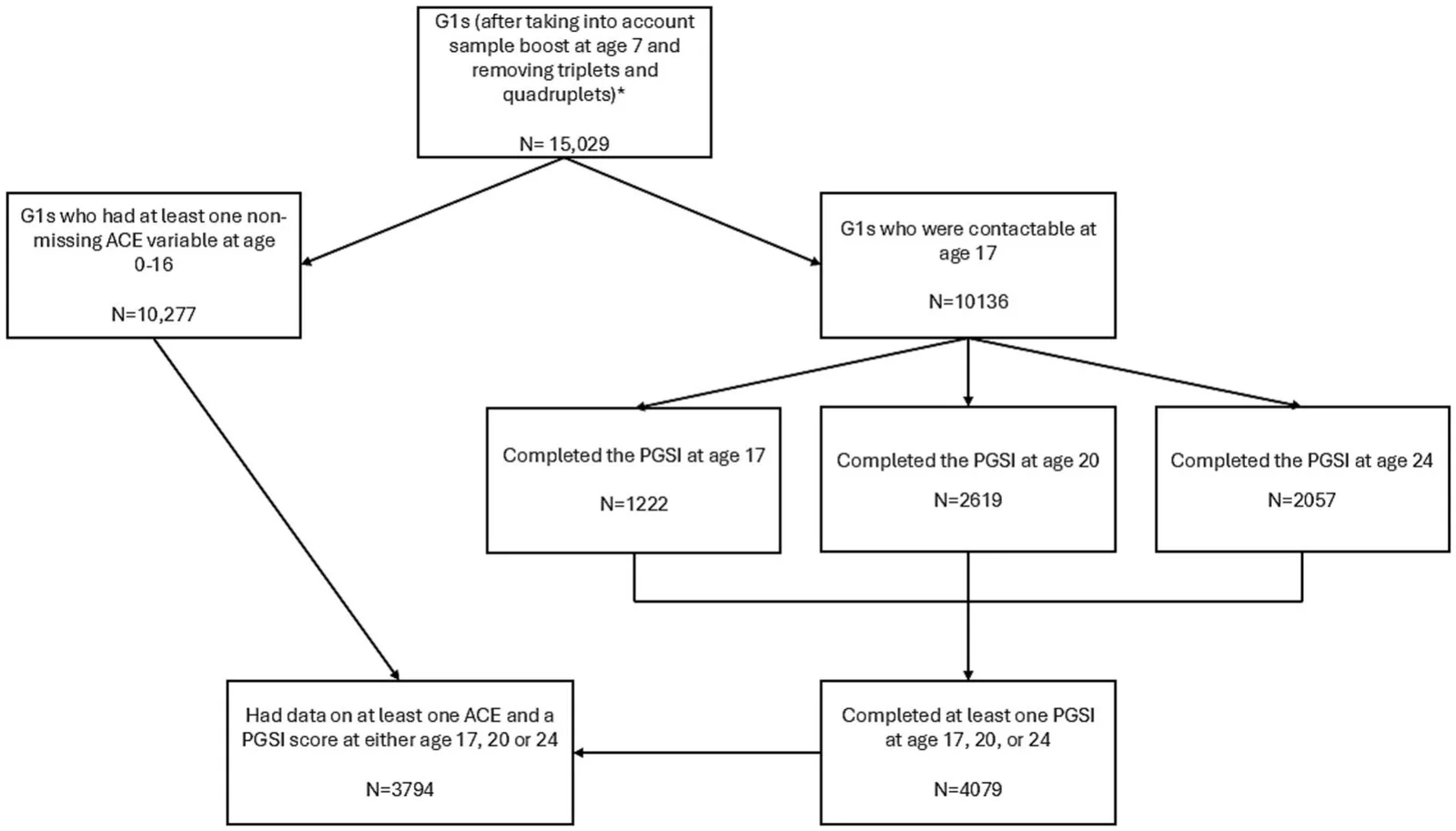
Flowchart of study participants. *Throughout, numbers are participants with non-missing sex. ACEs = Adverse childhood experiences; G1s = Offspring of the recruited pregnancies at baseline; PGSI = Problem gambling severity index.

### 3.2 Exposure: adverse childhood experiences

Individual binary variables (yes/no) for the following ACEs have been generated for the ALSPAC G1s previously: physical abuse, sexual abuse, emotional abuse, emotional neglect, bullying, violence between parents, household substance abuse, parental mental health problems or suicide, parent convicted of an offence, and parental separation, by Houtepen and colleagues (Houtepen et al., 2018). ACEs were not assessed using a single dedicated ACE questionnaire; instead, they variables were constructed from around 500 prospective questionnaire and clinic items that were parent-reported until the children were eight years old, and both parent- and child-reported until age 16. Because no single informant provides a definitive measure of experiences of adversity, these ACE indicators were derived using all available prospective information from both parent and child reports, with the aim of maximising ascertainment of adversity across childhood. These constructs broadly correspond to the ‘classic’ ACEs commonly used in the literature and are consistent with those listed in the World Health Organisation (WHO) Adverse Childhood Experiences International Questionnaire (ACE-IQ) (Lereya et al., 2015; World Health Organisation, 2020). Further details are provided in Houtepen et al (Houtepen et al., 2018). To both account for likely high levels of co-occurrence of different types of ACEs for individuals, and to explore a potential dose-response relationship, a total ACE ‘score’ was generated by summing the total number of different ACEs between 0 and 16 years (i.e. the score could range from 0 to 10). ACE counts were highly positively skewed; treating them as a continuous variable would imply linearity and produce unstable estimates at higher scores. We therefore categorised ACEs as 0, 1, 2–3, and 4+, following established practice (Hughes et al., 2017; Lacey & Minnis, 2019), and reflecting commonly used thresholds for low, moderate, and high cumulative adversity.

### 3.3 Outcome: gambling risk categories

The primary outcome was moderate-risk or problem gambling, measured using the shortened nine-item PGSI questionnaire (Ferris, 2001). The PGSI is a widely used and validated measure of gambling -related harm that was developed for use in general population surveys and has demonstrated good reliability and internal consistency in UK population samples (e.g., Cronbach's alpha = 0.89) (Gambling Commission, National Centre for Social Research, and University of Glasgow, 2024). Each item (of nine) is measured on a four-point scale with a maximum score of 27. Typically, researchers sum across the nine items and categorise the PGSI score as the following: 0 = ‘No risk of Problem Gambling’; 1–2 = ‘Low-risk of Problem Gambling’; 3–7 = ‘Moderate-risk of Problem Gambling’; 8+ = ‘Problem Gambling’. The number of participants meeting the threshold for problem gambling (scores of ≥8) was small at each age (*n* = 7, 25, and 30 at ages 17, 20, and 24, respectively. Therefore, we combined moderate-risk and problem gambling categories to create a binary outcome (a score of ≥3 vs. 0–2), consistent with other UK population surveys where high-severity cases are uncommon (Gambling Commission et al., 2024; NHS Digital, 2021), to improve model stability while retaining the distinction between no/low-risk and moderate-risk/problem gambling. To capture earlier stages of gambling risk that may precede moderate-risk gambling in young adulthood, we additionally examined an outcome defined as PGSI ≥1 (i.e. not 'no risk').

### 3.4 Covariates

We adjusted for the covariates that we hypothesised to be potential confounders of the ACE-gambling relationship. These covariates were all recorded at the time of the mother's pregnancy or birth of the baby, and many represented family socio-economic background, a key potential confounder of the ACE-gambling association: biological sex (registered at birth; gender identity was not available from the data), parity (0, 1, 2+), highest of mother's and father's educational qualifications (A-level or higher vs. lower), highest of mother's and father's social class (NS-SEC category, III manual+ or not), and mother ownership/mortgage (yes/no; father's was not captured).

### 3.5 Missing data approach

We accounted for missing data using multiple imputation with chained equations. We fitted imputation models that included ACEs, PGSI categories (no risk; low; moderate; problem) at all time-points, all covariates included in the adjusted model and auxiliary variables (at time of mother's pregnancy/young person's birth: numeric variables for mother's age, child's birthweight, mother's Edinburgh Postnatal Depression Score (EPDS) and partner's EPDS; and binary variables for White ethnicity and mother smoking in pregnancy). We assumed data to be Missing At Random. Although this assumption cannot be verified directly, we considered it plausible because the imputation model included all study variables together with large amounts of information from auxiliary variables associated with the exposures, outcomes and missingness (Sterne et al., 2009; White et al., 2011). We fitted models separately for women and men, given likely different patterns of missingness by sex, and potential interactions between ACEs and sex, created twenty imputed datasets, and pooled estimates across imputed datasets using Rubin's rules (Rubin, 1987). We present estimates after multiple imputation within the main results, and estimates from the complete data set only within supplementary materials.

### 3.6 Statistical analysis

All data cleaning and statistical models were carried out in STATA v.17. Tables and figures were created using R v.4.3.1. We analysed PGSI outcomes at each of the three ages of 17, 20 and 24, separately, representing distinct stages of young adulthood. We considered that three were too few time-points to model within-person gambling trajectories or repeated-measures structures without imposing strong and unrealistic assumptions about developmental change.

Although our focus was on adjusted associations between ACEs and gambling, we also present crude associations within supplementary materials, for reference. We fitted binary logistic regressions to estimate the associations of individual ACEs and ACE scores, with moderate-risk+ gambling (vs. no-to-low-risk), as crude models, and adjusted for variables listed above under ‘*Covariates*’. We fitted 66 separate models [11 different ACE exposures (10 individual binary, one ACE score categorical), a binary outcome at three different time-points, each crude and adjusted]. We then repeated these models with the outcome of low-risk+ gambling, as a secondary outcome of interest.

Conclusions on the strength of evidence for associations were based on the point estimates of the estimated odds ratios (ORs) and their associated 95% confidence intervals (CIs). That is, we do not focus on ‘statistical significance’, as recommended by the statistical community.(Amrhein et al., 2019).

To assess whether relationships between ACEs and gambling differed between men and women (i.e. whether sex modified the effect of ACEs on gambling outcomes), plausible given the ACEs and gambling literature (Allami et al., 2021; White & Kaffman, 2019), we initially included an interaction term between sex and each ACE exposure in analytic models (Knol & VanderWeele, 2012). Most of these interaction terms had high *p*-values (median across 36 *p*-values =0.46, inter-quartile range = 0.28 to 0.76). Therefore, in our final analyses and reporting we do not stratify models by sex.

To estimate the extent to which ACEs accounted for moderate-risk or problem gambling (anyone with a PGSI score of 3+) in young adults, population attributable fractions (PAFs) were calculated based on relative risks approximated to estimated adjusted odds ratios (aORs). The PAFs can be interpreted as the maximum proportion of cases of at least moderate-risk gambling that could be attributed to the specific ACE. PAFs were estimated using the formula described in **Supplementary Box S2**.

### 3.7 Ethics statement

Ethical approval for the study was obtained from the ALSPAC Ethics and Law Committee and the Local Research Ethics Committees (further details here: https://www.bristol.ac.uk/media-library/sites/alspac/documents/governance/Research_Ethics_Committee_approval_references.pdf). Informed consent for the use of data collected via questionnaires and clinics was obtained from participants following the recommendations of the ALSPAC Ethics and Law Committee at the time.

## 4 Results

### 4.1 Participants

A total of 3794 participants had data on at least one ACE and had completed the PGSI at either ages 17, 20, or 24 years (2079 female, 1715 male) (Table 1). Most participants were White. Around 82% of participants' maternal homes were owned or purchased with a mortgage, and around half of the participants' parents had A-level qualifications. The median EPDS score in participants' mothers was 6, with half having a score of 3 to 9 (representing from none to mild depression). Compared with the originally recruited ALSPAC cohort, participants were more likely to be White (95% vs. 93%), female (55% vs. 49%), and socioeconomically more advantaged (e.g. home ownership 82% vs. 69%).

**Table 1:** Breakdown of characteristics for participants with observed ACE and PGSI score, n (%) unless otherwise stated^a^.

	Study sample (*N* = 3794)		Original ALSPAC cohort (*N* = 14,904)	
**Ethnic group**				
White	3597	(94.8)	13,973	(93.0)
Other	197	(5.2)	1056	(7.0)
**Sex**				
Female	1715	(45.2)	7683	(51.1)
Male	2079	(54.8)	7346	(48.9)
**Maternal home ownership**				
Mortgaged/owned	667	(17.8)	4609	(31.1)
Rented or housing association	3080	(82.2)	10,219	(68.9)
**Social class (maternal)**				
I-III	538	(16.2)	2621	(21.4)
IV-V	2783	(83.9)	9611	(78.5)
Social class (paternal)				
I-III	1676	(44.1)	7507	(50.0)
IV-V	2335	(55.9)	7521	(50.0)
**Parity**				
0	1753	(48.5)	6430	(45.8)
1	1268	(35.1)	4832	(34.4)
2+	604	(16.7)	2785	(19.8)
**Maternal smoking in pregnancy**				
No	3180	(84.0)	11,480	(76.7)
Yes (1 or more cigarettes)	606	(16.0)	3490	(23.3)
**Mother's highest educational qualification**				
<A-level	2090	(55.1)	10,005	(66.6)
A-level+	1704	(44.9)	5024	(33.4)
**Father's highest educational qualification**				
<A-level	1473	(41.6)	6763	(51.1)
A-level+	2062	(58.3)	6438	(48.6)
**Mother age at delivery: median (interquartile range)**	29	(26 to 32)	28	(25 to 31)
**Mother Edinburgh postnatal depression score: median (interquartile range)**	6	(3 to 9)	7	(3 to 10)
**Father Edinburgh postnatal depression score: median (interquartile range)**	3	(1 to 6)	4	(2 to 7)
**Adverse Childhood Experience (age 0–16)**				
**ACE score (number of types experienced)**				
0	780	(20.6)	2680	(17.8)
1	1022	(26.9)	3853	(25.6)
2–3	1350	(35.6)	5539	(36.9)
4+	642	(16.9)	2957	(19.7)
**Type of ACE**				
Physical abuse	702	(18.5)	2261	(15.0)
Sexual abuse	175	(4.6)	497	(3.3)
Emotional abuse	707	(18.6)	2840	(18.9)
Emotional neglect	694	(18.3)	3152	(21.0)
Bullying	913	(24.1)	3691	(24.6)
Violence between parents	749	(19.7)	3327	(22.1)
Household substance abuse	424	(11.2)	2186	(14.5)
Parental mental health problems or suicide	1612	(42.5)	7042	(46.9)
Parent convicted of an offence	288	(7.6)	1384	(9.2)
Parental separation	1039	(27.4)	5128	(34.1)
**PGSI gambling categories at age 17**				
No risk of problem gambling (score 0)	2802	(73.8)	10,660	(70.9)
Low-risk of problem gambling (score 1–2)	757	(20.0)	3283	(21.8)
Moderate-risk of problem gambling (score 3–7)	208	(5.5)	926	(6.2)
Problem gambling (score ≥8)	27	(0.7)	159	(1.1)
**PGSI gambling categories at age 20**				
No risk of problem gambling (score 0)	2691	(70.9)	10,102	(67.2)
Low-risk of problem gambling (score 1–2)	836	(22)	3621	(24.1)
Moderate-risk of problem gambling (score 3–7)	226	(6.0)	1086	(7.2)
Problem gambling (score ≥8)	41	(1.1)	220	(1.5)
**PGSI gambling categories at age 24**				
No risk of problem gambling (score 0)	2932	(77.3)	11,130	(74.1)
Low-risk of problem gambling (score 1–2)	611	(16.1)	2648	(17.6)
Moderate-risk of problem gambling (score 3–7)	184	(4.8)	913	(6.1)
Problem gambling (score ≥8)	67	(1.8)	338	(2.2)

a These descriptive statistics have been derived in each of the imputed datasets and averaged as per ‘Rubin’'s rules’ (D. B. Rubin, Multiple imputation for non-response in surveys, Wiley, 1987).

The prevalence of ACEs was high, with over three-quarters of ALSPAC participants having at least one ACE (Table 1). The most frequently reported ACE was parental mental health problems or suicide, affecting 43% of participants, whereas the least reported ACE was sexual abuse, experienced by 5% of participants.

PGSI categories varied across ages. Across all three ages (17, 20, and 24 years), 71–77% were categorised as having no-risk of problem gambling, 16–22% were low-risk, 5–6% were moderate-risk, and 1–2% were categorised as problem gambling.

### 4.2 Association between ACEs and at least moderate-risk of problem gambling (PGSI ≥ 3)

Overall, there was little evidence of associations between most ACEs and at least moderate-risk of problem gambling. The main exception was sexual abuse, for which consistent positive associations were observed, although confidence intervals were wide. A majority of ACEs had point estimates in the direction of increased odds of at least moderate-risk gambling at ages 17, 20, and 24, with those exposed having up to 2.03 times the odds of moderate-risk+ gambling as those unexposed, but most effects generally small (26/30 estimates were positive ORs, median of 24 estimates 1.31, interquartile range [IQR] 1.21 to 1.51) (Fig. 2). See **Supplementary Table S1** for crude (unadjusted) ORs and CIs.

**Fig. 2:**
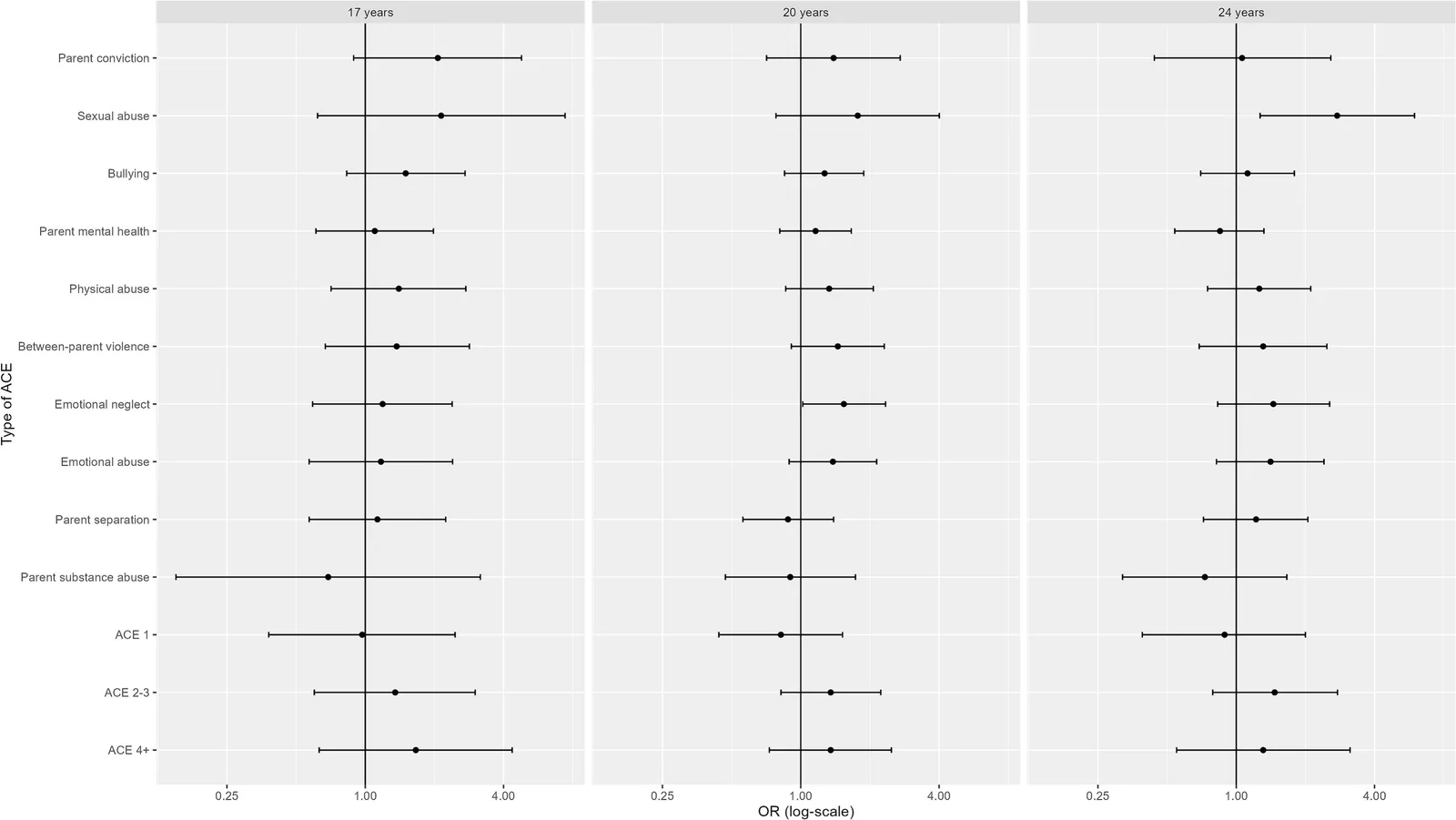
Adjusted odds ratios and confidence intervals for the relationship between Adverse Childhood Experiences and at least moderate-risk of problem gambling at ages 17, 20, and 24^a^ a. Exact values underlying this figure are provided in **Supplementary Table S2.**

After adjusting estimates for background socioeconomic factors, experiencing sexual abuse, physical abuse, between-parent violence, or emotional neglect were indicatively associated with higher odds of living with moderate-risk+ gambling at all age-points (25/30 positive aORs, median 1.37, IQR 1.19 to 1.45, with wide CIs; Fig. 2**, Supplementary Table S2**). Sexual abuse yielded the largest point estimates (aOR at age 17: 2.14 [0.62 to 7.41]; age 20: 1.77 [0.78 to 2.07]; age 24: 2.75 [1.27 to 5.97]).

In the case of parental conviction, bullying, and parental mental health problems, point estimates indicated higher odds of moderate-risk+ gambling at ages 17 and 20 (aORs ranged 1.10 to 2.07), but not at age 24 (0.88 to 1.06) (Fig. 2**, Supplementary Table S2**).

For number of ACEs, there were positive associations between 2–3 or 4+ ACEs vs. no ACEs, but with point estimates being similar between 2 and 3 and 4+ ACEs (aORs ranged 1.31 to 1.66), and little evidence for a dose-response relationship (Fig. 2**, Supplementary Table S2**).

### 4.3 Findings for low-risk+ of problem gambling compared to moderate-risk+ (PGSI ≥ 1)

Findings were broadly similar when the outcome threshold was lowered to at least low-risk of problem gambling (PGSI score ≥1; Fig. 3, **Supplementary Tables S3 and S4**). Sexual abuse and emotional neglect remained positively associated with gambling risk across all ages, while parental conviction, bullying, and parental mental health problems were associated with low-risk+ gambling at ages 17 and 20 but not 24. There was some weak evidence for a dose-response relationship with increasing number of ACEs category. PAFs were generally small, which ranged up to 8% for emotional neglect at age 24 **(Supplementary Table S5)**.

**Fig. 3:**
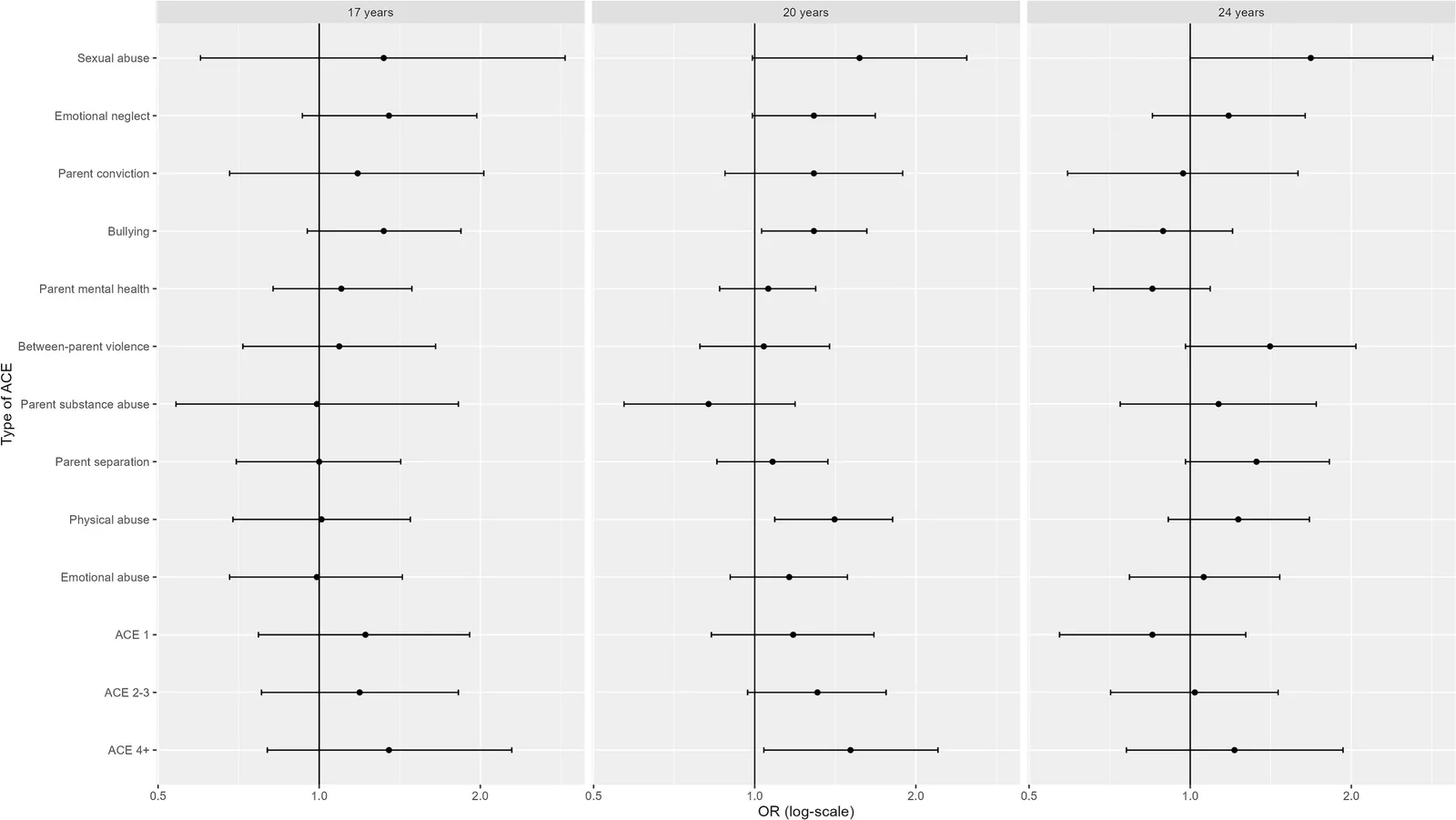
Adjusted odds ratios and confidence intervals for the relationship between Adverse Childhood Experiences and at least low-risk of problem gambling at ages 17, 20, and 24^a^. a. Exact values underlying this figure are provided in **Supplementary Table S4.**

### 4.4 Population attributable fractions of ACEs for moderate-risk+ gambling (PGSI≥ 3)

The PAFs, which represent the proportion of gambling cases attributable to ACEs, were low for all ACEs, with wide CIs (Table 2). The highest PAFs were for bullying and emotional neglect, where bullying accounted for 11%, 6%, and 3% of cases of at least moderate-risk of problem gambling at ages 17, 20, and 24 (CIs ranging −12% to 20%) and emotional neglect accounted for 4% to 9% (−16% to 15%), reflecting that PAFs represent not just the size of the aOR, but also the prevalence of the ACE in question (bullying and emotional neglect were considered present for 24% and 18% of the study sample; Table 1).

**Table 2:** Population Attributable Fractions (PAFs) for individual Adverse Childhood Experiences and at least moderate-risk of problem gambling at age 17, 20 and 24 years.

**Adverse Childhood Experience**	**Age 17**		**Age 20**		**Age 24**	
Physical abuse	7.2	(−10.2 to 16.0)	5.4	(−3.7 to 11.3)	4.8	(−7.6 to 12.3)
Sexual abuse	3.8	(−4.4 to 6.2)	2.1	(−1.4 to 3.7)	5.7	(1.9 to 7.5)
Emotional abuse	3.1	(−15.4 to 12.1)	6.3	(−2.8 to 12.1)	6.6	(−5.0 to 13.4)
Emotional neglect	3.6	(−15.6 to 13.1)	9.3	(0.5 to 15.2)	7.4	(−4.9 to 14.3)
Bullying	10.8	(−6.4 to 20.3)	6.2	(−5.0 to 13.8)	2.8	(−11.6 to 11.7)
Violence between parents	5.9	(−10.9 to 14.1)	7.1	(−2.2 to 12.9)	5.6	(−10.5 to 14.1)
Household substance abuse	−4.5	(−56.9 to 6.8)	−1.0	(−10.7 to 4.0)	−3.3	(−18.9 to 3.6)
Parental mental health problems or suicide	4.1	(−27.8 to 21.9)	6.1	(−10.6 to 17.7)	−7.4	(−34.6 to 9.9)
Parent convicted of an offence	7.1	(−1.6 to 10.9)	2.6	(−3.8 to 5.9)	0.5	(−11.7 to 5.6)
Parental separation	3.6	(−23.5 to 17.2)	−3.1	(−18.5 to 6.6)	5.6	(−12 to 16.0)

## 5 Discussion

Using prospective data from a large UK birth cohort, we observed suggestive positive but imprecise associations between several ACEs, particularly sexual abuse, physical abuse, and between-parent violence, and at least moderate-risk of problem gambling into young adulthood. There was little evidence for an increased number of different ACEs increasing the likelihood of moderate-risk or problem gambling. Population attributable fractions suggested that certain ACEs might account for up to 12% of cases of at least moderate-risk of problem gambling, but these estimates were highly uncertain due to wide confidence intervals.

### 5.1 Strengths and limitations

The main strengths of this study were prospective ACE measurement, reducing likelihood of recall bias, and repeated gambling outcomes. Consistency of associations across ages provided more robust evidence for a relationship between sexual abuse, physical abuse, between-parent violence and at least a moderate-risk of problem gambling. However confidence intervals were wide and often spanned the null. Across almost all models, the width of the confidence intervals means that the precision of our estimates is limited, and the observed associations should therefore be interpreted as tentative rather than definitive. Multiple imputation minimised the impact of partially missing data and allowed us to include nearly 4000 participants.

We captured ACEs through a comprehensive measure (captured using around 500 variables across childhood and adolescence). Binary ACEs indicators were derived pragmatically from the available information on that construct and so there was variation between the individual ACEs in terms of what time periods were covered, and who the ACE was reported by (Houtepen et al., 2018). It is feasible that response accuracy is differential between those with different ACEs due to the different thresholds for identification. For example, parental criminal conviction or separation are less likely to be susceptible to poor recall than emotional neglect. Further, ACEs such as emotional neglect at earlier time-points were parent-reported and thus may be under-reported due to social desirability bias. Such misclassification of ACEs would result in an under-estimate of positive associations; disproportionate misclassification between different types of ACEs would distort relative associations. We do not recommend ranking of ACEs in this study by magnitude of odds ratios, which slightly altered between moderate-risk+ and low-risk+ outcomes – nevertheless it is likely that sexual abuse is the most strongly associated with later gambling outcomes. Similarly, there is unlikely to be an association between emotional abuse and gambling outcomes at ages 17–24, given point estimates were close to the null at all three time-points for both risk thresholds. We found little evidence of a dose–response relationship between the number of ACEs and gambling risk, as point estimates for 2–3 and 4+ ACEs were similar and confidence intervals were wide. The weak evidence for a dose-response relationship, combined with somewhat stronger associations for specific adversities such as sexual abuse, may suggest that particular forms of adversity are more relevant to gambling vulnerability than cumulative ACEs alone. This pattern is consistent with developmental models that posit distinct pathways linking different forms of adversity to later behavioural outcomes (McLaughlin et al., 2014), rather than a purely cumulative-risk process. However, limited statistical power and measurement heterogeneity in ACE ascertainment may have attenuated any underlying dose–response pattern.

Although gambling was measured using a widely accepted and validated measure, the PGSI, there is still potential for reporting bias. Responses could be influenced by social desirability and therefore potential under-capture of gambling risk outcomes, which would again result in under-estimates of the positive association.

The generalisability of the findings may be limited by ALSPAC participant demographics, as the study sample were relatively affluent and majority White. Although the cohort originally enrolled in pregnancy was broadly representative of the British population at the time, differential attrition from ALSPAC has resulted in those who were socially disadvantaged being more likely to be lost to the study over time. Both ACEs and problem gambling are socially patterned, therefore we would hypothesise that any associations from this study are likely to be an underestimate (Felitti et al., 1998). However, the proportion of people experiencing ACEs and problem gambling within our study are within the range of previous prevalence estimates (Ford et al., 2019; Public Health England & Office for Health Improvement and Disparities, 2023). Further, previous methodological research in ALSPAC has found the effect of socially patterned loss-to-follow-up on risk estimates to be minimal (Howe et al., 2013). As our primary interest was in associations between ACEs and gambling outcomes, rather than estimating population prevalences, it is likely that the strongest associations in the general population are for sexual abuse, physical abuse, and between-parent violence.

### 5.2 Comparison with previous studies

Our point estimates for outcomes at multiple time-points were broadly in the same direction as previous cross-sectional studies linking sexual abuse to gambling harm (Allami et al., 2021; Bristow et al., 2022; Dennie et al., 2023; Poole et al., 2017), although the imprecision in our estimates means alignment should be interpreted cautiously. Other ACEs showed inconsistent associations: for example, an association was found between parental conviction, bullying, or parental mental health problems and at least moderate-risk of problem gambling at ages 17 and 20 but not 24, which correspond with previous findings of long-term effects of ACEs manifesting at different timepoints for other outcomes (Crick et al., 2022). However, the high levels of imprecision, and the large number of models fitted as part of this study increased the likelihood of false positives. Replication in a larger sample size than the current study would likely require cross-cohort studies and meta-analyses.

Although our study did not examine mediating mechanisms, the patterns of associations observed for sexual abuse, physical abuse, and between-parent violence are broadly compatible with theoretical accounts that conceptualise problem gambling as arising partly from emotional vulnerability and adverse developmental experiences (Blaszczynski & Nower, 2002).

### 5.3 Implications for policy, practice, and research

This study provides tentative longitudinal evidence that supports, but does not confirm, a possible relationship between sexual abuse and problem gambling, something key to address given statements in scoping reviews for national policy of insufficient evidence (Public Health England & Office for Health Improvement and Disparities, 2023). The findings also support a conceptualisation of gambling harms as arising from broader developmental and social contexts rather than solely from individual decision-making. Their long-term impact on gambling risk highlights the need for systemic responses rather than individual blame. Recognising the role of childhood adversity in gambling behaviour underscores the importance of policy interventions that go beyond individual-level solutions, such as stricter gambling regulations, consumer protections, and increased investment in harm prevention and treatment strategies.

If childhood adversity contributes to later gambling vulnerability, this suggests that prevention of gambling harms may benefit from a life-course approach that addresses early environmental and familial risk factors alongside gambling-specific interventions. Given relatively small adjusted ORs and small PAFs of ACEs for moderate-risk or problem gambling, it is likely that gambling behaviours are driven by a complex network of risk factors, of which ACEs are a part. ACE experience alone is likely to have limited utility for identifying young people at greatest risk of gambling-related harm, and prevention strategies would need to address multiple modifiable factors alongside ACEs and trauma (Sharman et al., 2019). Prevention and early intervention efforts may benefit from focusing on risk factors more proximal to gambling behaviour, alongside broader efforts to reduce childhood adversity.

These findings are in line with a body of evidence suggesting long-term potentially wide-ranging impacts of child maltreatment and between-parent violence into young adulthood, where there is already evidence for links with other adverse health behaviours and outcomes, such as smoking, substance misuse, and further violence (Bellis et al., 2019). Therefore, adding to the list of wider health and societal benefits of primary prevention interventions of ACEs (Asmussen et al., 2020; Gunstone & Gosschalk, 2020). Preventing ACEs through family support programmes, improved access to mental health services, and targeted social policies may have long-term benefits for reducing gambling-related harm (Mayer & Thursby, 2012; Moxey et al., 2026). The pattern of associations, albeit imprecise, is consistent with calls for trauma-informed approaches in gambling treatment (Moxey et al., 2026), including ACE screening and tailored psychological support.

Stronger evidence of the positive associations identified here, either through combination in meta-analysis, or replication in other large longitudinal datasets, are needed. Following such work, there would be a need to better understand timing and possible mechanisms for the relationships between ACEs and gambling. There was weak evidence for associations at some time-points and not others (either only earlier or later time-points), for bullying and parental conviction. Even later time-points could be explored when available from the ongoing data collection in ALSPAC, to provide evidence to generate hypotheses about how particular ACEs manifest as later adverse outcomes in particular.

## 6 Conclusions

The findings of our study could have important implications for both public health and gambling policy. The UK Government's Gambling Act Review White Paper 2023 concluded that there was insufficient evidence to establish ACEs as a predictor of gambling problems (Public Health England & Office for Health Improvement and Disparities, 2023). Our study provides suggestive but imprecise longitudinal evidence of a positive association between certain ACEs and at least moderate risk of problem gambling, consistent with developmental models that conceptualise childhood adversity as an early-life determinant of later gambling vulnerability, and reinforcing the need to consider childhood adversity in gambling prevention and treatment efforts. Given the high prevalence of ACEs and their well-documented impact on behavioural and mental health outcomes (Bellis et al., 2019; Felitti et al., 1998), early intervention strategies that reduce experiences of childhood adversity could mitigate, among other outcomes, gambling-related harm. Given the uncertainty around these estimates, further research is needed to clarify whether these associations exist and, if so, through what mechanisms they might arise. Overall, although many point estimates were in the direction of increased risk, the wide and often null-spanning confidence intervals mean that these findings should be viewed as exploratory and hypothesis-generating rather than conclusive.

## Statement on use of Generative Artificial Intelligence (AI)

When making updates to the manuscript, the senior author used Copilot version bizchat.20260316.49.2 to improve the clarity and conciseness of the manuscript's wording. All ideas, analyses, and interpretation are the authors' own.

## CRediT authorship contribution statement

**Emily Patterson:** Writing – review & editing, Writing – original draft, Formal analysis. **Raffaello Rossi:** Writing – review & editing, Supervision, Conceptualization. **Hannah Sallis:** Writing – review & editing, Supervision, Conceptualization. **Emma Dennie:** Writing – review & editing. **Laura D. Howe:** Writing – review & editing. **Alan Emond:** Writing – review & editing. **Annie Herbert:** Writing – review & editing, Writing – original draft, Supervision, Formal analysis, Data curation, Conceptualization.

## Ethics declaration

Written informed consent to take part in the study and to publish the article has been obtained from all participants or their legal representatives. The privacy rights of participants have been observed.

This study was performed in compliance with relevant laws, regulatory frameworks and guidelines where the research took place. Ethics committee approval was not required under relevant laws and institutional guidelines. All new data collection studies conducted with the ALSPAC cohort require ethical approval. Further details are provided here: https://www.bristol.ac.uk/alspac/researchers/research-ethics/. We carried out analysis of anonymised secondary data.

## Funding

The UK 10.13039/501100000265Medical Research Council and Wellcome (Grant ref.: 217065/Z/19/Z) and the 10.13039/501100000883University of Bristol provide core support for ALSPAC. A comprehensive list of grants funding is available on the ALSPAC website (http://www.bristol.ac.uk/alspac/external/documents/grant-acknowledgements.pdf). Specific funding for the ALSPAC Gambling Study was supplied by GambleAware and the University of Bristol. At the time of carrying out this work, HS, ED, LDH, and AH worked at UK Medical Research Council Integrative Epidemiology Unit at the University of Bristol (Grant ref.: MC_UU_00032/7). AH is funded by a Sir Henry Wellcome postdoctoral fellowship (Grant ref. 224114/Z/21/Z). This publication is the work of the authors and the first author will serve as guarantor for the contents of this paper.

## Declaration of competing interest

The authors declare that they have no known competing financial interests or personal relationships that could have appeared to influence the work reported in this paper.

## Data Availability

The data that has been used is confidential. ALSPAC data access is through a system of managed open access. The ALSPAC access policy (https://www.bristol.ac.uk/media-library/sites/alspac/documents/researchers/data-access/ALSPAC_Access_Policy.pdf) describes the process of accessing the data and samples in detail, and outlines the costs associated with doing so. You can submit your research proposal for consideration by the ALSPAC Executive Committee. You will receive a response within 10 working days to advise you whether your proposal has been approved. If you have any other questions about accessing data, please email alspac-data@bristol.ac.uk.

## Footnotes

1 Problem Gambling is the official term used in the Problem Gambling Severity Index (PGSI),but there is debate about the individualising nature of this term, with a suggested move to ‘people who experience gambling problems’.
