# Supplementary material for: Longitudinal associations between adverse childhood experiences and moderate-risk to problem gambling in young adulthood: A prospective UK cohort study
Source: Addict Behav Rep. 2026 Oct 22;24:100764. doi: 10.1016/j.abrep.2026.100764 (PMC13763127; doi:10.1016/j.abrep.2026.100764)
Supplement: Supplementary file 1 [file mmc1.docx]

**Supplementary materials**

**Box S1. Additional details about ALSPAC and the current study sample.**

Pregnant people with an expected delivery date between 1^st^ April 1991-31^st^ December 1992, and who lived in Avon, UK were invited to join the cohort. 20,248 pregnancies were identified as being eligible and the initial number of pregnancies enrolled was 14,541. Of the initial pregnancies, there was a total of 14,676 foetuses, resulting in 14,062 live births and 13,988 children who were alive at one year of age. When the oldest children were approximately seven years of age, an attempt was made to bolster the initial sample with eligible cases who had failed to join the study originally. The total sample size for analyses using any data collected after the age of seven is therefore 15,447 pregnancies, resulting in 15,658 foetuses. Of these 14,901 children were alive at one year of age, the index offspring or G1 cohort.

ALSPAC data were collected and managed using REDCap (Research Electronic Data Capture) hosted at the University of Bristol. REDCap is a secure, web-based software platform designed to support data capture for research studies (Harris et al., 2009). Please note that the study website contains details of all the data that is available through a fully searchable data dictionary and variable search tool (<http://www.bristol.ac.uk/alspac/researchers/our-data/>).

As part of rules for accessing ALSPAC data, triplets and quadruplets were excluded from analyses, as this was extremely rare (n=13) and these individuals are considered too identifiable (Boyd et al., 2013). In the original ALSPAC cohort, there were 195 sets of twins; after applying all other exclusion criteria, there were 40 sets of twins (1% of the study sample) in the analytical sample – we posit that clustering within these families would have minimal impact on estimates.

At the age 17 clinic during 2008-2010 (5^th^-95^th^ centiles of age: 17-18), 5,217 ALSPAC participants completed a research clinic visit at which they were invited to fill in an additional questionnaire about gambling behaviours (including PGSI questions), to which 3,829 consented. At the ages 20 and 24 clinics (in 2012-2013 and 2016, respectively), 9,067 and 9215 cohort participants were still enrolled and contactable in ALSPAC; of these participants 2,625 and 2,758 completed online or postal questionnaires.

Of the original 15029 G1s, 10277 (68%) had at least one non-missing ACE variable across age 0-16 (**Fig. 1**); 3616 (24%) had non-missing data across all the ACEs studied. Of the 10,136 G1s who were contactable at age 17, 4079 (40%), 1505 (15%) and 342 (3%) responded to the PGSI at at least one time-point, two time-points, and all three time-points, respectively.

**Box S2. Formula for estimating Population Attributable Fractions**

We used the following formula:

$$\hat{PAF}=\frac{\hat{P}(A=1|Y=1)(e^{\hat{\beta}_{1}}-1)}{e^{\hat{\beta}_{1}}}\times100$$

where $\hat{P}(A=1|Y=1)$ is the estimated prevalence of the ACE in question among those with a positive outcome (moderate-risk+ gambling) and $e^{\hat{\beta}_{1}}$ is the estimated aOR of moderate-risk+ gambling at ages 17, 20, and 24 years for each of the 10 ACEs (Ferguson et al., 2019). PAFs provide an alternative interpretation of aORs in the context of this study’s ACE prevalence, as they are a function of both aOR and prevalence. In other words, PAFs indicate the proportion of gambling cases in this population that could be attributed to each ACE - suggesting the potential number of cases that might be prevented if that ACE were eliminated.

**Table S1. Crude estimates for the relationship between Adverse Childhood Experiences and moderate-risk+ gambling at ages 17, 20, and 24^a^**

|  | **Age 17** | | | **Age 20** | | | **Age 24** | | |
| --- | --- | --- | --- | --- | --- | --- | --- | --- | --- |
| **Adverse Childhood Experience** | **OR** | **(CI)** | **p-value** | **OR** | **(CI)** | **p-value** | **OR** | **(CI)** | **p-value** |
| Physical abuse | 1.59 | (0.85 to 2.96) | 0.14 | 1.34 | (0.87 to 2.07) | 0.19 | 1.32 | (0.78 to 2.25) | 0.31 |
| Sexual abuse | 1.89 | (0.60 to 5.98) | 0.28 | 1.01 | (0.47 to 2.18) | 0.97 | 2.03 | (1.03 to 4.02) | 0.04 |
| Emotional abuse | 1.10 | (0.53 to 2.27) | 0.80 | 1.23 | (0.81 to 1.89) | 0.34 | 1.31 | (0.79 to 2.18) | 0.29 |
| Emotional neglect | 1.36 | (0.70 to 2.67) | 0.37 | 1.53 | (1.02 to 2.30) | 0.04 | 1.49 | (0.91 to 2.44) | 0.12 |
| Bullying | 1.54 | (0.89 to 2.68) | 0.12 | 1.41 | (0.97 to 2.06) | 0.07 | 1.25 | (0.79 to 1.98) | 0.34 |
| Violence between parents | 1.31 | (0.63 to 2.73) | 0.48 | 1.28 | (0.80 to 2.06) | 0.31 | 1.29 | (0.73 to 2.27) | 0.38 |
| Household substance abuse | 0.91 | (0.35 to 2.36) | 0.84 | 0.81 | (0.43 to 1.52) | 0.51 | 0.91 | (0.43 to 1.89) | 0.80 |
| Parental mental health | 1.02 | (0.61 to 1.71) | 0.94 | 1.10 | (0.78 to 1.57) | 0.58 | 0.95 | (0.62 to 1.47) | 0.83 |
| Parent convicted of an offence | 2.00 | (0.89 to 4.49) | 0.09 | 1.29 | (0.67 to 2.46) | 0.45 | 1.17 | (0.53 to 2.54) | 0.70 |
| Parental separation | 1.20 | (0.65 to 2.23) | 0.56 | 0.79 | (0.49 to 1.27) | 0.34 | 1.27 | (0.79 to 2.02) | 0.32 |
| ACE score (vs. 0) |  |  |  |  |  |  |  |  |  |
| 1 | 0.96 | (0.44 to 2.12) | 0.92 | 0.82 | (0.47 to 1.42) | 0.47 | 1.24 | (0.69 to 2.21) | 0.47 |
| 2-3 | 1.53 | (0.51 to 4.57) | 0.45 | 0.89 | (0.52 to 1.54) | 0.68 | 1.05 | (0.55 to 2.02) | 0.87 |
| 4+ | 2.11 | (0.55 to 8.07) | 0.28 | 0.91 | (0.41 to 2.00) | 0.81 | 1.25 | (0.50 to 3.09) | 0.63 |

a. This table displays the results of 11 separate logistic regression models, pooled across 20 imputed datasets.

ACE = Adverse childhood experience; CI = Confidence interval; OR = Odds ratio

**Table S2. Adjusted estimates for the relationship between Adverse Childhood Experiences and moderate-risk+ gambling at ages 17, 20, and 24^a^**

|  | **Age 17** | | | **Age 20** | | | **Age 24** | | |
| --- | --- | --- | --- | --- | --- | --- | --- | --- | --- |
| **Adverse Childhood Experience** | **OR** | **(CI)** | **p-value** | **OR** | **(CI)** | **p-value** | **OR** | **(CI)** | **p-value** |
| Physical abuse | 1.40 | (0.71 to 2.74) | 0.33 | 1.33 | (0.86 to 2.07) | 0.21 | 1.26 | (0.75 to 2.11) | 0.38 |
| Sexual abuse | 2.14 | (0.62 to 7.41) | 0.23 | 1.77 | (0.78 to 4.01) | 0.17 | 2.75 | (1.27 to 5.97) | 0.01 |
| Emotional abuse | 1.17 | (0.57 to 2.40) | 0.66 | 1.38 | (0.89 to 2.14) | 0.15 | 1.41 | (0.82 to 2.41) | 0.22 |
| Emotional neglect | 1.19 | (0.59 to 2.39) | 0.63 | 1.54 | (1.02 to 2.34) | 0.04 | 1.45 | (0.83 to 2.55) | 0.19 |
| Bullying | 1.50 | (0.83 to 2.72) | 0.18 | 1.27 | (0.85 to 1.88) | 0.24 | 1.12 | (0.70 to 1.79) | 0.65 |
| Violence between parents | 1.37 | (0.67 to 2.84) | 0.39 | 1.45 | (0.91 to 2.31) | 0.12 | 1.31 | (0.69 to 2.48) | 0.41 |
| Household substance abuse | 0.69 | (0.15 to 3.17) | 0.63 | 0.90 | (0.47 to 1.73) | 0.75 | 0.73 | (0.32 to 1.66) | 0.45 |
| Parental mental health | 1.10 | (0.61 to 1.98) | 0.74 | 1.16 | (0.81 to 1.66) | 0.42 | 0.85 | (0.54 to 1.32) | 0.46 |
| Parent convicted of an offence | 2.07 | (0.89 to 4.79) | 0.09 | 1.39 | (0.71 to 2.71) | 0.34 | 1.06 | (0.44 to 2.58) | 0.89 |
| Parental separation | 1.13 | (0.57 to 2.24) | 0.73 | 0.88 | (0.56 to 1.39) | 0.59 | 1.22 | (0.72 to 2.05) | 0.46 |
| ACE score (vs. 0) |  |  |  |  |  |  |  |  |  |
| 1 | 0.97 | (0.38 to 2.46) | 0.94 | 0.82 | (0.44 to 1.52) | 0.53 | 0.89 | (0.39 to 2.00) | 0.77 |
| 2-3 | 1.35 | (0.60 to 3.01) | 0.46 | 1.35 | (0.82 to 2.23) | 0.24 | 1.47 | (0.79 to 2.76) | 0.23 |
| 4+ | 1.66 | (0.63 to 4.36) | 0.30 | 1.35 | (0.73 to 2.48) | 0.34 | 1.31 | (0.55 to 3.13) | 0.54 |

a. This table displays the results of 11 separate ordinal logistic regression models, pooled across 20 imputed datasets. Each model was adjusted for the following variables: sex, maternal home ownership status, parity, maternal smoking in pregnancy, mother and father’s highest educational qualification, household social class, child ethnic background, mother at age of delivery, and mother and father’s depression scores. Evidence of association is based on 95% CIs.

ACE = Adverse childhood experience; CI = Confidence interval; OR = Odds ratio

**Table S3. Crude estimates for the relationship between Adverse Childhood Experiences and low-risk+ gambling at ages 17, 20, and 24^a^**

|  | **Age 17** | | | **Age 20** | | | **Age 24** | | |
| --- | --- | --- | --- | --- | --- | --- | --- | --- | --- |
| **Adverse Childhood Experience** | **OR** | **(CI)** | **p-value** | **OR** | **(CI)** | **p-value** | **OR** | **(CI)** | **p-value** |
| Physical abuse | 1.08 | (0.75 to 1.57) | 0.68 | 1.36 | (1.05 to 1.76) | 0.02 | 1.22 | (0.91 to 1.65) | 0.19 |
| Sexual abuse | 1.09 | (0.49 to 2.39) | 0.84 | 1.23 | (0.80 to 1.89) | 0.35 | 1.36 | (0.84 to 2.20) | 0.22 |
| Emotional abuse | 0.90 | (0.62 to 1.31) | 0.60 | 1.07 | (0.84 to 1.35) | 0.60 | 1.00 | (0.72 to 1.38) | 0.99 |
| Emotional neglect | 1.45 | (1.00 to 2.10) | 0.05 | 1.25 | (0.97 to 1.61) | 0.08 | 1.20 | (0.87 to 1.65) | 0.26 |
| Bullying | 1.35 | (0.99 to 1.84) | 0.05 | 1.32 | (1.05 to 1.66) | 0.02 | 0.93 | (0.69 to 1.24) | 0.60 |
| Violence between parents | 1.10 | (0.72 to 1.67) | 0.66 | 0.99 | (0.76 to 1.28) | 0.92 | 1.40 | (1.03 to 1.90) | 0.03 |
| Household substance abuse | 1.11 | (0.67 to 1.84) | 0.69 | 0.83 | (0.59 to 1.16) | 0.27 | 1.13 | (0.78 to 1.63) | 0.52 |
| Parental mental health | 1.10 | (0.83 to 1.46) | 0.50 | 1.02 | (0.84 to 1.24) | 0.86 | 0.87 | (0.69 to 1.12) | 0.28 |
| Parent convicted of an offence | 1.21 | (0.69 to 2.15) | 0.51 | 1.28 | (0.89 to 1.84) | 0.18 | 1.00 | (0.62 to 1.61) | 0.99 |
| Parental separation | 1.08 | (0.76 to 1.53) | 0.68 | 1.09 | (0.86 to 1.39) | 0.46 | 1.35 | (1.02 to 1.78) | 0.04 |
| ACE score (vs. 0) |  |  |  |  |  |  |  |  |  |
| 1 | 1.18 | (0.75 to 1.85) | 0.47 | 1.13 | (0.85 to 1.51) | 0.41 | 0.87 | (0.60 to 1.25) | 0.45 |
| 2-3 | 1.29 | (0.85 to 1.94) | 0.23 | 1.25 | (0.94 to 1.65) | 0.13 | 1.07 | (0.76 to 1.49) | 0.70 |
| 4+ | 1.41 | (0.86 to 2.30) | 0.17 | 1.38 | (0.98 to 1.94) | 0.06 | 1.20 | (0.81 to 1.77) | 0.36 |

a. This table displays the results of 11 separate ordinal logistic regression models, pooled across 20 imputed datasets.

ACE = Adverse childhood experience; CI = Confidence interval; OR = Odds ratio

**Table S4. Adjusted estimates for the relationship between Adverse Childhood Experiences and low-risk+ gambling at ages 17, 20, and 24^a^**

|  | **Age 17** | | | **Age 20** | | | **Age 24** | | |
| --- | --- | --- | --- | --- | --- | --- | --- | --- | --- |
| **Adverse Childhood Experience** | **OR** | **(CI)** | **p-value** | **OR** | **(CI)** | **p-value** | **OR** | **(CI)** | **p-value** |
| Physical abuse | 1.01 | (0.69 to 1.48) | 0.95 | 1.41 | (1.09 to 1.81) | 0.01 | 1.23 | (0.91 to 1.67) | 0.18 |
| Sexual abuse | 1.32 | (0.60 to 2.88) | 0.49 | 1.57 | (0.99 to 2.49) | 0.06 | 1.68 | (1.00 to 2.84) | 0.05 |
| Emotional abuse | 0.99 | (0.68 to 1.43) | 0.94 | 1.16 | (0.90 to 1.49) | 0.26 | 1.06 | (0.77 to 1.47) | 0.71 |
| Emotional neglect | 1.35 | (0.93 to 1.97) | 0.12 | 1.29 | (0.99 to 1.68) | 0.07 | 1.18 | (0.85 to 1.64) | 0.33 |
| Bullying | 1.32 | (0.95 to 1.84) | 0.10 | 1.29 | (1.03 to 1.62) | 0.03 | 0.89 | (0.66 to 1.20) | 0.44 |
| Violence between parents | 1.09 | (0.72 to 1.65) | 0.68 | 1.04 | (0.79 to 1.38) | 0.78 | 1.41 | (0.98 to 2.04) | 0.07 |
| Household substance abuse | 0.99 | (0.54 to 1.82) | 0.98 | 0.82 | (0.57 to 1.19) | 0.29 | 1.13 | (0.74 to 1.72) | 0.57 |
| Parental mental health | 1.10 | (0.82 to 1.49) | 0.52 | 1.06 | (0.86 to 1.30) | 0.61 | 0.85 | (0.66 to 1.09) | 0.20 |
| Parent convicted of an offence | 1.18 | (0.68 to 2.03) | 0.56 | 1.29 | (0.88 to 1.89) | 0.19 | 0.97 | (0.59 to 1.59) | 0.91 |
| Parental separation | 1.00 | (0.70 to 1.42) | 0.98 | 1.08 | (0.85 to 1.37) | 0.54 | 1.33 | (0.98 to 1.82) | 0.07 |
| ACE score (vs. 0) |  |  |  |  |  |  |  |  |  |
| 1 | 1.22 | (0.77 to 1.91) | 0.40 | 1.18 | (0.83 to 1.67) | 0.35 | 0.85 | (0.57 to 1.27) | 0.44 |
| 2-3 | 1.19 | (0.78 to 1.82) | 0.42 | 1.31 | (0.97 to 1.76) | 0.08 | 1.02 | (0.71 to 1.46) | 0.91 |
| 4+ | 1.35 | (0.80 to 2.29) | 0.27 | 1.51 | (1.04 to 2.20) | 0.03 | 1.21 | (0.76 to 1.93) | 0.43 |

a. This table displays the results of 11 separate ordinal logistic regression models, pooled across 20 imputed datasets. Each model was adjusted for the following variables: sex, maternal home ownership status, parity, maternal smoking in pregnancy, mother and father’s highest educational qualification, household social class, child ethnic background, mother at age of delivery, and mother and father’s depression scores. Evidence of association is based on 95% CIs.

ACE = Adverse childhood experience; CI = Confidence interval; OR = Odds ratio

**Table S5. Population Attributable Fractions (PAFs) for individual Adverse Childhood Experiences and low-risk+ gambling at age 17, 20 and 24 years**

| **Adverse Childhood Experience** | **Age 17** | | **Age 20** | | **Age 24** | |
| --- | --- | --- | --- | --- | --- | --- |
|  | **PAF** | **(CI)** | **PAF** | **(CI)** | **PAF** | **(CI)** |
| Physical abuse | 0.2 | (-8.6 to 6.3) | 6.0 | (1.8 to 9.4) | 4.0 | (-2.1 to 8.6) |
| Sexual abuse | 1.1 | (-3.0 to 2.9) | 2.0 | (-0.1 to 3.3) | 2.6 | (0.0 to 4.2) |
| Emotional abuse | -0.3 | (-9.0 to 5.7) | 2.7 | (-2.3 to 6.6) | 1.1 | (-5.5 to 5.9) |
| Emotional neglect | 5.5 | (-1.6 to 10.5) | 4.9 | (-0.3 to 8.8) | 3.0 | (-3.5 to 7.7) |
| Bullying | 6.8 | (-1.6 to 12.8) | 6.1 | (0.8 to 10.3) | -2.7 | (-11.2 to 3.6) |
| Violence between parents | 1.5 | (-7.1 to 7.2) | 0.8 | (-5.2 to 5.3) | 6.9 | (-0.5 to 12) |
| Household substance abuse | -0.1 | (-9.6 to 5.1) | -2.2 | (-7.6 to 1.5) | 1.4 | (-4.1 to 5.0) |
| Parental mental health | 4.2 | (-10.0 to 14.8) | 2.2 | (-7.1 to 9.8) | -7.1 | (-20.7 to 3.4) |
| Parent convicted of an offence | 1.4 | (-4.3 to 4.7) | 1.9 | (-1.1 to 4.0) | -0.2 | (-5.3 to 2.9) |
| Parental separation | -0.1 | (-12.5 to 8.6) | 2.1 | (-5.2 to 7.8) | 7.9 | (-0.7 to 14.2) |

CI = Confidence interval
